# An optoionic hydrogel with UV-regulated ion conductivity for reprogrammable iontronics: Logic processing and image sensing

**DOI:** 10.1126/sciadv.adn0439

**Published:** 2024-06-12

**Authors:** Jiehao Chen, Jiahe Huang, Yuhang Hu

**Affiliations:** ^1^ The George W. Woodruff School of Mechanical Engineering, Georgia Institute of Technology, Atlanta, GA 30332, USA.; ^2^ The School of Chemical and Biomolecular Engineering, Georgia Institute of Technology, Atlanta, GA 30332, USA.

## Abstract

The development of smart hydrogels capable of actively controlling ion conductivity is of paramount importance for iontronics. Most current work in this field focuses on enhancing the hydrogels’ ion conductivity. Few successes have been seen in achieving spatial regulation of ion flow through external control. Among various controls, light gives the best spatial and temporal resolution for practical iontronic applications. However, developing hydrogels that can generate drastic ion concentration change upon photoirradiation for tunable conductivity is challenging. Very few molecules can enable photoion generation, and most of them are hydrophobic and low quantum yield. Here, we present an optoionic hydrogel that uses triphenylmethane leuconitrile (TPMLN) for ultraviolet-regulated ion conductivity. Through postpolymerization TPMLN synthesizing, we can incorporate high concentration of the hydrophobic TPMLN in hydrogels without compromising the hydrogel’s mechanical integrity. Upon light irradiation, the hydrogel’s local conductivity can change an unprecedented 10-fold. We also demonstrated soft optoionic devices that are capable of logic processing and photo imaging.

## Teaser

An optoionic hydrogel is designed with spatial programmable conductivity by UV dynamic patterning for reconfigurable iontronics.

## INTRODUCTION

Many critical biological processes involve electrical signal transduction such as sensing, healing, communication, actuation, and more (*1*–*3*). Different from current electronics that conduct electrons through rigid inorganic components, biology uses ions to conduct electric signals through a cascade of coordinated electro-chemo-mechanical processes hosted by integrated systems of soft and wet biocomponents. There exist intrinsic mismatches between current electronics and biological systems in both electrical, mechanical, and chemical properties and performance. In recent years, there have been noticeable successes in developing iontronic devices that have better mechanical, electrical, and chemical compatibilities to interface with the soft and ion-conductive biological tissues, opening up avenues for health care applications (*4*–*6*), bionic systems (*7*), and biohybrid robotics (*8*).

Among the recent developments, a widely used material for iontronic applications is hydrogel. It consists of cross-linked polymer networks and solvents. It has high stretchability (*9*, *10*), good biocompatibility (*11*), and versatile stimuli-responsive properties (*12*–*16*). To meet the basic requirements for iontronic applications, efforts have been given to improve the hydrogels’ mechanical integrity and ion conductivity (*17*–*19*), which have enabled various devices and capabilities such as the stretchable ionic cables that resemble the biological axons (*20*), touch sensing devices, artificial skins (*21*, *22*), and bioactivity transducers (*23*, *24*). Efforts have also been made to incorporate stimulus responsiveness into hydrogel iontronics, such as glucose, (*25*), nitrogen oxide, or ammonia responsiveness (*26*), as well as pH (*27*, *28*), solvent polarity (*29*), mechanical load (*30*, *31*), temperature (*32*, *33*), electrical field (*21*, *34*), magnetic field (*35*), and photothermal responsiveness (*36*). These hydrogels use chemical reactions or physical input to change the ion-polymer network interaction. Their conductivities are still at a lower end even at their “on-state,” limiting their ability to carry a larger current to directly drive circuit components, so they are predominately used as sensors in these demonstrations rather than full-fledged iontronic components. In addition, the spatial resolution of these controls is relatively low and not suitable for precise control. In contrast, biological systems can modulate ionic signals at the cellular level with exceptionally high spatial and temporal resolutions and thus can realize functions that are unmatched by synthetic iontronic systems such as tactile and image sensing and logic processing (*37*, *38*).

To develop next-generation iontronics that mimic the information processing capability of biological systems and can handle multidimensional inputs as current electronics, it is crucial to develop hydrogels that can regulate local ion behavior with high spatial resolutions. One effective approach to achieve this is to incorporate photoionization reactions into hydrogels. By inducing a photoinduced change in ion mobility, it becomes possible to achieve continuous and localized control over the iontronic properties within the material. A noticeable example of this is the demonstration of photoswitchable conductivity in a supramolecular hydrogel consisting of one host (chitosan) and two guest molecules (azobenzene and C8 alkyl sulfate) (*39*). Upon light activation, the interaction between azobenzene and chitosan weakens, allowing chitosan to preferentially interact with C8 alkyl sulfate ions and reduce their mobility. However, the ion conductivity in the “on” state remains low because of the limited mobility of the large C8 alkyl sulfate ions, and because the tunability of the conductivity of this material relies on a delicate balance of intramolecular interaction kinetics among the three molecules, the tuning range of conductivity is less than two times, posing challenges in practical applications.

## RESULTS

### Optoionic hydrogel preparation with postpolymerization triphenylmethane leuconitrile synthesizing

**Fig. 1.**
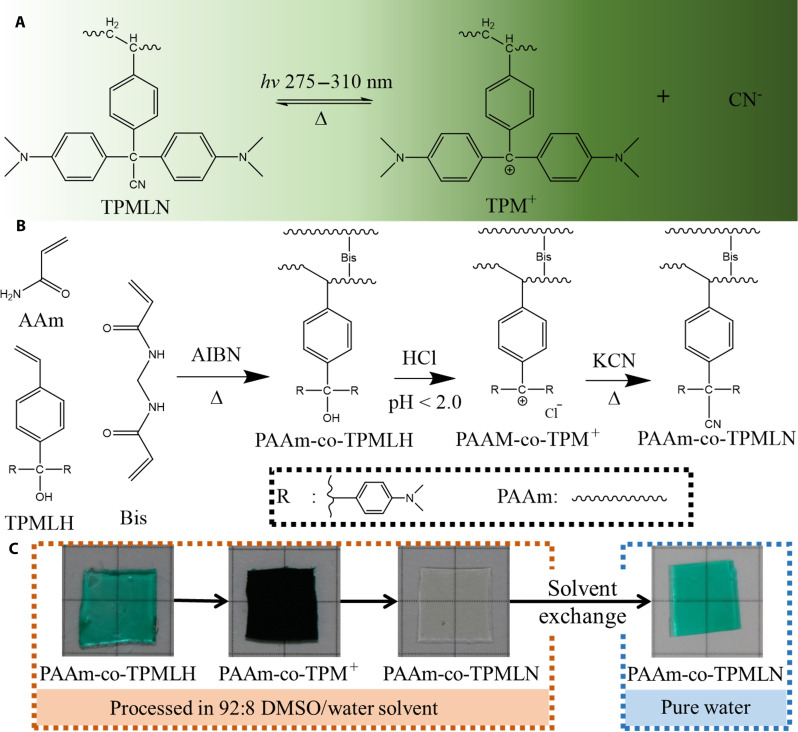
Mechanism of TPMLN photocleavage. (**A**) The UV-activated (275 to 310 nm) reversible photoionization reaction of TPMLN molecule. (**B**) To incorporate a high concentration of TPMLN into a PAAm hydrogel, TPMLH (more soluble) is first copolymerized with PAAm and then converted to TPMLN in acidic environment. (**C**) Examples of hydrogel sample after each conversion step and final solvent exchange step. Grid size, 5 mm.

Here, we developed a photoionizable hydrogel that exhibits high spatial and temporal control and a more than an order of magnitude changes of conductivity upon ultraviolet (UV) irradiation and use it to realize special capabilities including logic processing and photo imaging. The current widely used photosensitive molecules for making photoresponsive hydrogels are azobenzene and spiropyran. Under light irradiation, the azobenzene molecule undergoes reversible trans-cis photoisomerization that changes the molecule’s size, shape, and polarity, while the spiropyran undergoes photoisomerization to generate a zwitterionic structure. These conformational changes of the molecules change their intramolecular/electrostatic interactions with the surrounding liquid and, in turn, change the ion mobility inside the hydrogels. However, these processes do not directly generate free ions, and thus, the change of the hydrogels’ ion conductivities is very limited. Instead, in this work, we use photocleavage reactions to directly induce ion concentration changes in a hydrogel. Specifically, we use triphenylmethane leuconitrile (TPMLN) molecules. Upon UV exposure, TPMLN generates a cyanide anion with high mobility (*40*) (Fig. 1A). The counter ion (TPM^+^ cation) remains attached to the main chain, and the electrostatic force ensures that the cyanide ion stays in the vicinity of the pendent TPM^+^. Upon removal of the UV light, the cyanide ion quickly recombines with the counter ion TPM^+^ to return to an electrically neutral state. In comparison with other photoionizable molecules such as triphenylmethane leucohydroxide (TPMLH) or *o*-nitrobenzaldehyde, TPMLN dissociation has better reversibility, higher photoefficiency, and faster dissociation-reassociation kinetics (*41*) enabling better performance for large amplitude and dynamic control of hydrogel’s ion conductivity. In addition, TPMLN is less sensitive to environmental factors such as temperature and pH, ensuring more stable performance under various operating conditions. The TPMLN-incorporated hydrogel has been studied for its photoinduced swelling behaviors. In this study, it is used for generating phototunable conductivities.

Directly incorporating a high concentration of TPMLN into PAAm hydrogel has many challenges. Because the molecule is hydrophobic, it requires the use of a less polar solvent during the hydrogel preparation process. The presence of a less polar solvent like tetrahydrofuran (THF) can adversely affect the quality of polymerization and result in poor mechanical properties of the hydrogel (*42*–*44*). In addition, even using a less polar solvent, the maximum amount of TPMLN that can be incorporated is still limited, which is not sufficient to generate a substantial change in conductivity. In addition, TPMLN tends to precipitate into needle-like crystals when polymer network forms, creating microcracks and further weakening the hydrogel. To overcome the challenges associated with the direct incorporation of large amounts of TPMLN into the hydrogel, we introduce a postpolymerization conversion method (Fig. 1B). We first copolymerize another triphenylmethane leuco derivative molecule, TPMLH, with acrylamide (AAm) and *N, N′*-methylenebisacrylamide (Bis) to form the polyacrylamide (PAAm)-co-TPMLH hydrogel in dimethyl sulfoxide (DMSO)/deionized (DI) water environment. Compared with TPMLN, TPMLH has much higher solubility in more polar solvents and will not precipitate into needle-like crystals during polymerization. After the PAAm-co-TPMLH hydrogel is prepared, it is swollen in a mixture of 0.01 M hydrogen chloride (HCl) and 0.1 M potassium cyanide (KCN) in DMSO/DI water and put at 80°C environment. Under these conditions, the TPMLH will be converted into TPMLN through a two-step reaction (Fig. 1B). The final product should be near transparent, indicating complete recombination of TPMLN (Fig. 1C and fig. S1). Subsequently, the PAAm-co-TPMLN hydrogel is washed multiple times with DI water to remove DMSO, HCl, KCN, and other reactants. After the solvent exchange with DI water that has a higher polarity, a small amount of TPMLN will dissociate and make the sample a light green appearance (*45*). The resulting hydrogel contains a high concentration of TPMLN attached to the main chain. Following this protocol, hydrogel samples are prepared with fixed 3 M Am, 0.030 M Bis concentration, and varying TPMLN concentration from 0 to 0.090 M. Compared with direct polymerization of TPMLN with PAAm, the postconverted hydrogel remains higher in shear modulus (7.45 kPa versus 4.60 kPa) and stretchability (135% versus 49%) even at very high TPMLN concentration (fig. S2). The detailed synthesis procedure can be found in Materials and Methods.

### Conductivity response of the optoionic hydrogel

**Fig. 2.**
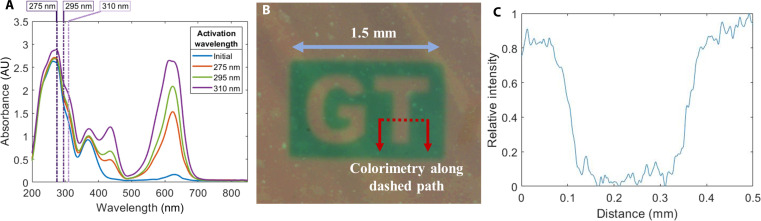
Quantification of the optoionic conductivity of PAAM-co-TPMLN hydrogel. (**A**) Absorption spectrum of a hydrogel sample with different activation wavelengths at its maximum activation state. (**B**) Hydrogel sample with patterned UV light activation showing a “GT” logo with well controlled spatial resolution. (**C**) Color changing profile measurement of the red marked line in (B).

The PAAm-co-TPMLN hydrogel is responsive to multiple-wavelengths light in UV band. Along with the photodissociation reaction, notable color change can be observed as an indication of the reaction progress. For different wavelengths of UV light, the efficiency of photon absorption and the light attenuation properties can lead to different responses within the same hydrogel system at different wavelengths. To determine the optimal wavelength for activating TPMLN, we conducted UV-visible (UV-vis) absorption spectroscopy from 200 to 850 nm (Fig. 2A). Three hydrogel samples with the 0.010 M TPMLN were prepared in the form of flat disks measuring ϕ10 mm by 0.2 mm and sealed inside custom quartz cuvettes (fig. S3). Each sample is then subjected to UV exposure from a collimated UV light-emitting diode (LED) source operating at a wavelength of 275, 295, or 310 nm (Marktech Optoelectronics UV LEDs). The UV LEDs were driven by a constant current source, and the optical power was calibrated to 0.2 mW/cm^2^ using an optical power meter (Thorlabs PM16-401). Following the UV exposure for 3 min, the hydrogel samples were promptly transferred to the UV-vis spectrometer (Spectral Product SM245) equipped with the ASBP-DW-F-BAL light source to capture the absorption spectrum. This process allowed us to analyze the changes in absorption characteristics resulting from the UV-induced photodissociation of TPMLN in the hydrogel samples (fig. S3). As shown in Fig. 2A, the hydrogel initially exhibits strong absorption for light below 400 nm, along with a small absorption peak at 630 nm, which corresponds to the color of the inactivated hydrogel. Following UV exposure, the dissociation of TPMLN leads to the emergence of two absorption peaks in the visible light range, specifically at 440 and 630 nm. Although the hydrogel exhibits better absorption for 275-nm UV light, the activation efficiency is notably lower compared to the 310-nm light source with the same irradiance. By analyzing the color gradient of the hydrogel through thickness captured from an upright microscope, 295-nm light can only penetrate 160 μm for 50% activation, whereas 310-nm light can penetrate up to 280 μm in a similar time frame (fig. S4). Therefore, when activating a hydrogel with a bigger thickness, it is preferable to use a 310-nm light source for more efficient activation and improved penetration depth. To demonstrate the spatial control of the material, we quantified the width of the boundary between the light-activated region and the inactivated region. We used a dynamic photomask (Mightex Polygon 1000, Zeiss AxioScope A1, 310 nm, 0.2 mW/cm^2^) to project a UV pattern onto a 200-μm-thick sample (Fig. 2B). When UV exposure took place, a well-defined “GT” pattern emerged within the exposed region, displaying a distinct and sharp boundary. In the region that turns green, the TPMLN molecule is dissociated and generates a mobile ion. The spatial resolution is characterized by measuring the hydrogel color profile across the activated and inactivated regions (Fig. 2C). The measured transition zone width is around 60 to 70 μm.

**Fig. 3.**
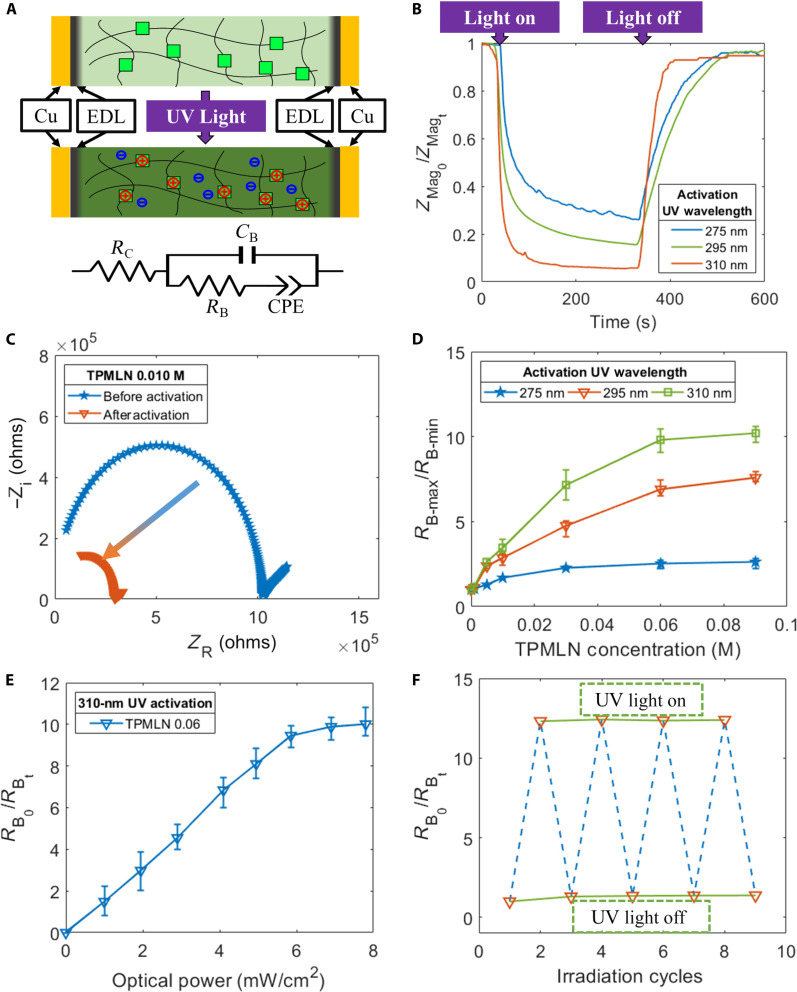
Quantification of the optoionic conductivity of PAAM-co-TPMLN hydrogel. (**A**) Measuring hydrogel ac impedance before and after UV activation. The circuit schematic shows the equivalent circuit used to fit the impedance measurement, with *R*_*C*_, *R*_*B*_, and *C*_*B*_ corresponding to the contact resistance, bulk resistance, and bulk capacitance. (**B**) Single-frequency ac impedance measures TPMLN activation and deactivation kinetics during and after UV irradiation. (**C**) Nyquist plots of ac-impedance measurements before and after UV activation. The *x* axis and *y* axis correspond to the real part and imaginary part of the measured impedance. (**D**) Relative change in resistance of a hydrogel strip with different TPMLN concentrations and activation wavelength at its maximum activation state. (**E**) Ratio between hydrogel’s initial bulk charge transfer resistance (*R*_*B*_0__) and postactivation bulk charge transfer resistance (*R*_*B*_*t*__) with different UV irradiance. (**F**) Hydrogel cyclic optoionic performance under alternating UV/dark conditions.

Alongside absorption spectroscopy, we conducted both single-frequency and multifrequency ac impedance spectroscopy to quantitatively evaluate the wavelength response of the PAAm-co-TPMLN hydrogel, as illustrated in Fig. 3. The hydrogel samples were prepared as 10-mm by 3-mm by 1-mm (length by width by thickness) pieces and securely positioned between two parallel copper electrodes (Fig. 3A and fig. S5). The ac impedance of each hydrogel sample was measured using a Keysight E4980A LCR meter. The complex form of the measured ac impedance illustrates the ac response of the system (Fig. 3A). In this experimental setup, an iontronic and electronic interface is established between the copper and hydrogel, forming an electrical double layer (EDL). These EDLs function as capacitors that are connected in series with the bulk polarization capacitance. At each electrode, electrochemical reactions can occur, showing a behavior similar to a constant phase element (CPE). The combined behavior of this system can be effectively represented through an equivalent circuit, as depicted in Fig. 3A (*46*). This circuit comprises various elements, namely *R*_*C*_ (contact resistance), *R*_*B*_ (bulk charge transfer resistance), *C*_*B*_ (bulk capacitance), and *CPE*, which collectively emulate the interactions within the system.

We used single-frequency ac impedance spectroscopy to analyze the dynamic response of the hydrogel subjected to the light change. The magnitude of the complex impedance (*Z*_Mag_) is plotted against time as illustrated in Fig. 3B. In this study, the samples with 0.010 M TPMLN were tested with 275-, 295-, or 310-nm UV light irradiations. The ac impedance of the interconnected copper-hydrogel configuration was measured under potentiostat mode, with an excitation frequency of 100 Hz and a peak-to-peak voltage of 100 mV. The change in impedance was tracked over time. When the UV light is on, an initial rapid change in impedance was observed, which subsequently reached a plateau. Upon removing the UV light, a reversed impedance changing pattern was observed. Notably, the utilization of 310-nm UV irradiation induced a more substantial resistance change at equilibrium, coupled with a swifter switching speed in contrast to the samples activated with 275- and 295-nm wavelengths.

To characterize the change of the hydrogel’s intrinsic conductivity after UV activation, we used multifrequency ac impedance spectroscopy and fit the result with an equivalent circuit model in ZView (tables S1 and S2) to retrieve the bulk charge transfer resistance *R*_*B*_. The hydrogel’s impedance is measured as a function of the input frequency ranging from 20 Hz to 2 MHz, with a peak-to-peak voltage of 100 mV. Figure 3C illustrates an example of the Nyquist plot obtained from the ac impedance measurements conducted on the 0.010 M TPMLN sample before and after activation with 310-nm UV light. Each point in the given plot represents the real and imaginary parts of complex impedance *Z* at a certain frequency. After activation, the “arc” diameter is notably reduced, indicating a reduction in bulk resistance.

Different wavelengths of UV light can have different activation efficiency. Figure 3D shows the relative conductivity change for samples with different TPMLN concentrations and activated with 275-, 295-, and 310-nm UV light at 5 mW/cm^2^ respectively. This change is defined as the ratio between the maximum bulk charge transfer resistance (*R*_*B-max*_) of the initial neutral hydrogel and the minimum *R*_*B*_ value of the fully activated hydrogel (*R*_*B-min*_). Consistent with the findings from the absorption spectral study, we observed similar responsiveness of the PAAm-co-TPMLN hydrogel to different UV wavelengths. Both 295- and 310-nm wavelength UV provided comparable activation efficiency, while 275-nm wavelength UV was less efficient in triggering the cleavage of cyanide ions from TPMLN. Regarding the TPMLN concentration, we observed that the tunable range of the hydrogel’s conductivity increased and reached a plateau as the TPMLN concentration increased. The plateau can be attributed to the substantial light attenuation caused by high TPMLN concentration and the equilibrium constraint of the reaction due to excessively generated cyanide ions. Overall, we observed a maximum reduction in resistivity of 92% with a TPMLN concentration of 0.090 M using 310-nm UV light. This demonstrates the substantial tunability of conductivity achieved in the optoionic hydrogel system.

The photodissociation reaction relies on the photon absorption of TPMLN. Continuous tuning of the hydrogel resistance is achieved by changing UV irradiance gradually. The ratio between the initial bulk resistance (*R*_*B*_0__) and the postactivation state (*R*_*B*_*t*__) is plotted as a function of input optical power from 0 to 8 mW/cm^2^ with a 1-mW/cm^2^ increment (Fig. 3E). The impedance is measured after at least 3 min of UV exposure until the impedance reading is stabilized. Because the reaction is reversible, the hydrogel conductivity can be reversibly switched by turning the LED on and off. The cyclic behavior of the hydrogel is shown in Fig. 3F with good repeatability.

### Optoionic hydrogel for logic processing

**Fig. 4.**
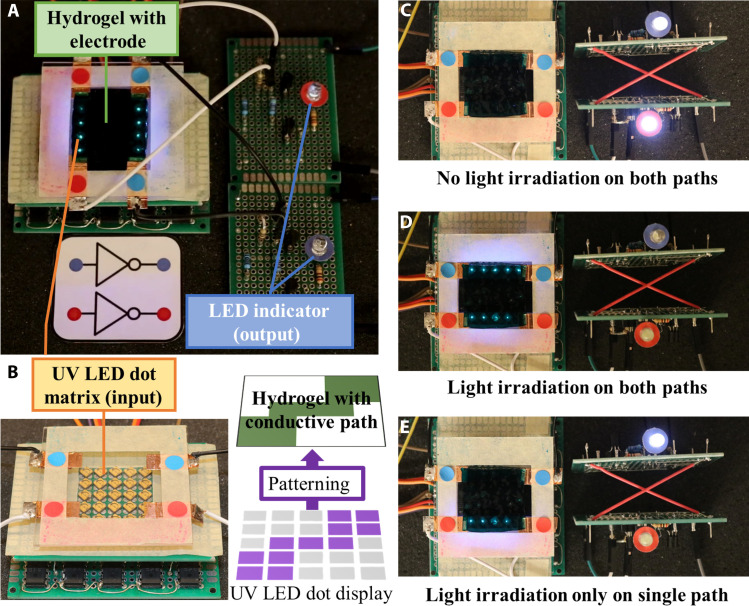
Using optoionic hydrogel to control LEDs in an analog circuit. (**A**) Photo of the circuit setup. (**B**) The LED array can selectively activate certain regions of the hydrogel. (**C**) When no UV irradiation input, both LEDs light up. (**D**) With UV irradiation input on both paths, both LEDs dim out. (**E**) With only one side exposed to UV irradiation, only one LED lights up.

The optoionic hydrogel can serve as a receptor, converting light inputs into ion concentration changes that are reflected as impedance variations in a circuit. Their high spatial resolution and extensive impedance tunability enable multiple receptors to be accommodated on a single hydrogel piece. To demonstrate its capabilities, we constructed an optoionic logic circuit, using the hydrogel as both a receptor and a processing unit. Figure 4A illustrates the setup of the logic processing unit, comprising two NOT gates integrated into a single PAAm-co-TPMLN hydrogel piece (0.060 M TPMLN concentration) (30 mm by 30 mm by 1 mm). Each logic circuit controls the dimming and illumination of a corresponding LED (marked as red/blue). Detailed information on the circuit design is available in Supplementary Text (fig. S6). The square-shaped hydrogel was placed on a quartz plate with four copper electrical contacts at each corner, allowing current to flow through two paths and control two LEDs (red-red and blue-blue). Beneath the hydrogel, a programmable UV LED array, controlled by a separate computer, selectively illuminated different regions of the hydrogel through the quartz plate (Fig. 4B). For instance, the red-red path was exposed to UV light, while the blue-blue path remained dark. To scale the current flow through the hydrogel to a safe operating range for the LEDs, a pair of amplifying PNP and NPN bipolar junction transistors were used. When the entire hydrogel was free of UV irradiation, the resistance of the hydrogel sheet was uniformly high, resulting in the illumination of both LEDs (Fig. 4C). Upon UV exposure to both the red and blue paths, their resistance decreased, causing LEDs A and B to dim (Fig. 4D). Turning off the UV LEDs on the blue path allowed TPMLN to gradually recombine to its neutral state, increasing the conductivity on the blue path and reactivating the corresponding LED. However, the other LED remained dimmed as the red path continued to be exposed to UV light (Fig. 4E). A complete video demonstration can be found in the Supplementary Materials (movie S1: NOT gate).

**Fig. 5.**
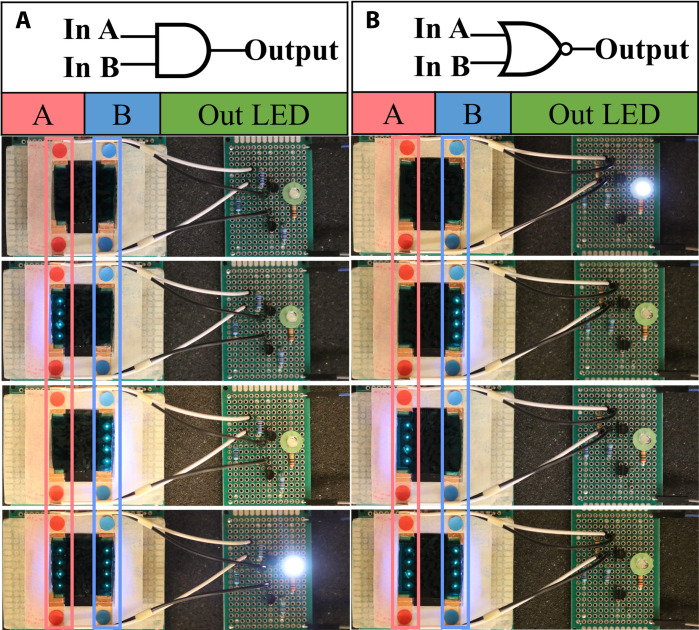
PAAm-co-TPMLN optoionic hydrogel for complex logic processing. An (**A**) AND gate and a (**B**) NOR gate is realized using the same piece of hydrogel with two separately controlled light paths. The on-off state of the output LED is controlled by two light inputs.

In addition to the NOT gate, we successfully implemented an AND gate and a NOR gate using the same piece of gel and the same setup with a modified circuit configuration. Detailed information on the circuit design can be found in Supplementary Text, and video demonstrations are available in movie S2 (AND gate) and movie S3 (NOR gate). In this setup, a single piece of hydrogel provides two conductive paths (red-red and blue-blue) that are connected to a specially configured circuit, controlling a single output LED. In comparison to the previous case where two designated paths are connected to two separate circuits, this is a more demanding design because the cross-talk between two paths through the middle portion of the hydrogel cannot be ignored. It requires the optoionic hydrogel to have a very large idle resistance and a notably smaller active resistance, so the nonactivated middle portion of the hydrogel sheet can act as an insulator to minimize the cross-talk between two designated paths. The same UV LED array is used as signal input. When configured as an AND gate, both sides of the UV LEDs must be in the ON state to activate the output LED (Fig. 5A). Conversely, when configured as a NOR gate, both sides of the UV LEDs must be in the OFF state to maintain the output LED in the ON state (Fig. 5B).

### Optoionic hydrogel as an artificial retina

**Fig. 6.**
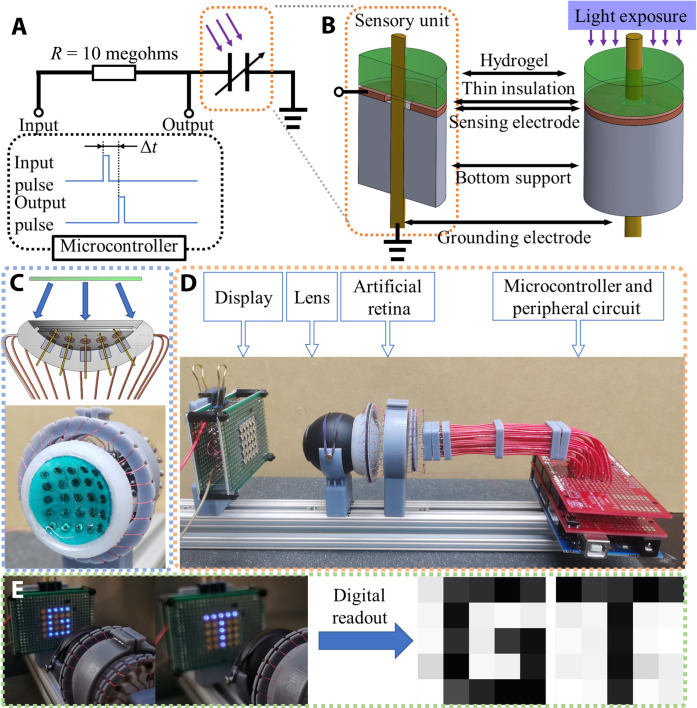
Using optoionic hydrogel as an artificial retina. (**A** and **B**) This image sensor is based on capacitive sensing where the hydrogel is used as the dielectric layer in the capacitor array. When hydrogel is UV activated, its dielectric property changes. The microcontroller can detect the capacity change and convert it to light intensity. (**C**) The hydrogel is soft and stretchable. We can conform this hydrogel layer onto an arbitrary curved surface. (**D**) The testing apparatus and (**E**) demonstration.

On the basis of the unique properties of the hydrogel, we also developed a system capable of “seeing” light through impedance sensing and generating graphical outputs. Specifically, we designed an artificial retina that can capture information from a UV LED array and recreate images using a MATLAB computer algorithm. In this setup, the optoionic hydrogel serves as the dielectric layer within a specially structured capacitor, rather than directly carrying current in the circuit. The dielectric constant of the hydrogel changes upon UV exposure because of the generation of mobile ions. Previous studies have shown a decreasing trend in polyelectrolyte hydrogel dielectric constant as the mobile ion species concentration increases (*47*). After UV irradiation, the cyanide ion concentration increases inside the hydrogel, rendering a reduction in capacitance in the corresponding light-sensing capacitor. In this artificial retina, each capacitor represents one sensory pixel and is connected to an Atmel Mega 2560 microcontroller through an external circuit (Fig. 6A and fig. S7). The capacitance of each capacitor can be measured on the basis of its charging-discharging characteristic time, which is related to the time lag between the input pulse from the microcontroller and the output voltage response across the capacitor. Therefore, the capacitance is correlated to light intensity through precalibration (fig. S8). Figure 6B shows the geometry of each capacitor. The sensing electrode is a top-insulated flat copper washer seated on a three dimensional printed plastic support (outer diameter, 3 mm; inside diameter, 1 mm). The grounding electrode is a needle wire protruding out from the middle hole of the washer electrode without making physical contact (0.4-mm diameter). The hydrogel is placed on top of the copper ring with an insulation layer in between. The needle electrode pokes through the hydrogel and makes direct contact with the hydrogel. The needle electrodes of all pixels are connected to common ground. The sensing electrodes are connected to individual I/O ports of the microcontroller (fig. S7). This special design makes the capacity only sensitive to localized ion concentration changes on the sensory ring electrode, minimizing cross-talk and improving imaging quality. Consequently, multiple closely packed sensory units are accommodated on a single piece of the hydrogel. In this design, each sensory array has a diameter of 3 mm and is arranged in a 5 × 5 array with 2-mm spacing (Fig. 6C). To mimic the curved surface of the human eye retina and enable powerful imaging capabilities within a compact form factor, we leveraged the stretchability and softness of the hydrogel to prepare the artificial retina in a curved shape. After assembling the capacitor electrode array into a curved silicone rubber encapsulation, a circular-shaped hydrogel sheet (TPMLN 0.060 M) was installed on top of the curved electrode array (Fig. 6C). Given the soft and stretchable nature of the hydrogel, the hydrogel sheet can elastically conform to the sensory array without wrinkling. For testing purposes, a plastic shell was attached in front of the artificial retina, which includes a convex lens. This plastic shell primarily serves to block scattered light, while the convex lens (UV fused silica Plano-Convex; diameter, 12.7 mm; *f* = 20.1 mm) focused the displayed information from the UV LED array onto the artificial retina (Fig. 6D). All signal cables were gathered using a customized circuit board adaptor and connected to the microcontroller. The raw capacitance readouts were transmitted to a computer for postprocessing into a 5 × 5-pixel grayscale image (Fig. 6E). This allows us to recreate the captured information and obtain visual representations of the sensed light patterns.

## DISCUSSION

In summary, we introduced an innovative approach for the preparation of high-performance optoionic hydrogels by in situ conversion of triphenylmethane derivatives after hydrogel polymerization. By copolymerizing a UV-sensitive photodissociable functional molecule, the hydrogel can actively change the local ion conductivity. The proposed postpolymerization TPMLN conversion method enables the homogeneous incorporation of a substantial amount of hard-to-dissolve TPMLN molecules into a PAAm hydrogel, improving the tuning range of its ion conductivity and preserving the mechanical integrity of the hydrogel. Through a reversible UV-activated photocleavage reaction, the hydrogel exhibits precise and remote modulation of conductivity and dielectric properties by generating free ions with high mobility.

With this unique hydrogel material, we demonstrated the potential of the optoionic hydrogel for biomimetic systems capable of conducting ionic signals and processing light stimuli into electrical signals. By constructing an information processing circuit around the optoionic hydrogel, we showcased its ability to process spatially applied UV stimuli and generate output information based on predefined logic patterns. Furthermore, we successfully developed an artificial retina consisting of 25 pixels using this optoionic hydrogel through capacitive sensing. The concept and applications of the optoionic properties presented in this study open previously unidentified avenues for the utilization of hydrogels in the fields of optoionics and bioelectronics. The ability to actively control ion flow and process light inputs in hydrogels expands their potential for a wide range of applications.

## MATERIALS AND METHODS

### Triphenylmethane derivative synthesize

Here, the TPMLH is first synthesized [4-bromo-*N, N*-dimethylaniline (97%, Sigma-Aldrich), *n*-butyllithium solution (2.5 M, Sigma-Aldrich), and methyl 4-vinylbenzoate (97%, Sigma-Aldrich)] by following the protocol presented by Jiang and co-workers (*48*). Anhydrous 4-bromo-*N, N*-dimethylaniline (4-BrDMA) is dissolved in THF (50 ml 0.54 M) under argon atmosphere and cooled to −78°C. *n*-Butyl lithium (*n*-BuLi) hexane solution (13 ml 2.5 M) was added drop by drop under constant stirring and cooling. The mixture is then left for 30 min for complete reaction. Dropwise methyl 4-vinylbenzoate THF solution (15 ml 0.9 M) is then added to the mixture under constant stirring at −78°C. The mixture is then left for reaction and warmed slowly to room temperature overnight under stirring. To extract the product, THF is spin evaporated under vacuum. The solid phase was then dissolved in diluted hydrochloric acid followed by the addition of sodium hydroxide solution (0.1 M) to pH 10.0. The precipitate is extracted using dichloromethane (DCM), then dried with anhydrous sodium sulfate. The crude product is obtained by vacuum evaporation of DCM and recrystallized in methanol and hexane to yield a pale-green solid precipitate. The precipitate is dried using vacuum oven at room temperature.

The mass spectrometry (MS) electrospray ionization (ESI) spectrum and the ^1^H-NMR spectrum results are as follows. ^1^H-NMR (DMSO-d6): 7.31 (d, 2H, m-H of PhCdC), 7.13 (d, 2H, o-H of PhCdC), 6.93 (d, 4H, o-H of NPh), 6.67 (dd, 1H, PhCHdC), 6.59 (d, 4H, m-H of NPh), 5.74 (d, 1H, cis-H of PhCHdCH2), 5.18 (d, 1H, trans-H of PhCHdCH2), 2.82 (s, 12H, -NCH3). MS ESI mass/charge ratio = 355.1982 found for C25H27N2+, theoretical value 355.22.

### Hydrogel preparation

To prepare this optoionic hydrogel, two major steps are involved. The polyacrylamide-co-triphenylmethane leucohydroxide (PAAm-co-TPMLH) polymer is first synthesized in an organic phase. Precursors of acrylamide (3 M, ≥98% Sigma-Aldrich), *N, N′*-methylenebisacrylamide (0.03 M, 99% Sigma-Aldrich), and TPMLH (0 ~ 0.090 M, as synthesized) are dissolved into 92% vol. DMSO (Sigma-Aldrich)–water mixture. Thermoactivated initiator 2,2′-azobis(2-methylpropionitrile) (98% Sigma-Aldrich) is added to initiate radical polymerization. The monomer solution (2 ml) is degassed under vacuum with agitation. The polymerization is made in a sandwiched glass mold in a hot water bath at 80°C for 4 hours. The resulting organo-gel is then thoroughly washed multiple times in hydrogen chloride solution (0.100 M), 92% vol. DMSO-water mixture solution until the solution color no longer changes. The gel (approx. 2 g) is then swelled under constant stirring at 70°C in hydrogen chloride solution (100 ml 0.010 M), 92% vol. DMSO-water mixture solution with additional potassium cyanide (0.1 M, ≥98% Sigma-Aldrich) added. The gel can be removed from the mixture solution once it becomes fully transparent. The resulting PAAm-co-TPMLN hydrogel is then thoroughly washed in DI water four times to remove HCl, KCN, and DMSO residual. Each time, the sample is immersed in a fresh DI water bath for 1 hour (*17*, *49*, *50*). The final PAAm-co-TPMLN hydrogel swollen in water should show a pale green color. The final hydrogels with 0- to 0.090-m TPMLN in equilibrium swelling are measured to have 2.1– to 4.5% wt polymer concentration. To verify the TPMLH to TPMLN conversion over different soaking steps, we performed Fourier transform infrared (FTIR) measurements on initial and final samples, as well as samples from intermediate steps. The hydrogel samples were dried and grounded into powers for FTIR testing. The FTIR results indicate a successful conversion of TPMLH to TPMLN in the postpolymerization conversion reaction (fig. S1).
