# Supplementary material for: An optoionic hydrogel with UV-regulated ion conductivity for reprogrammable iontronics: Logic processing and image sensing
Source: Sci Adv. 2024 Jun 12;10(24):eadn0439. doi: 10.1126/sciadv.adn0439 (PMC11168472; doi:10.1126/sciadv.adn0439)
Supplement: Supplementary file 1 — Supplementary Text Figs. S1 to S8 Tables S1 and S2 Legends for movies S1 to S3 [file sciadv.adn0439_sm.pdf]

Science Advances
AAAS

Supplementary Materials for

An optoionic hydrogel with UV-regulated ion conductivity for reprogrammable iontronics: Logic processing and image sensing

Jiehao Chen *et al*.

Corresponding author: Yuhang Hu, yuhang.hu@me.gatech.edu

Sci. Adv. **10**, eadn0439 (2024)
DOI: 10.1126/sciadv.adn0439

The PDF file includes:

Supplementary Text
Figs. S1 to S8
Tables S1 and S2
Legends for movies S1 to S3

Other Supplementary Material for this manuscript includes the following:

Movies S1 to S3

Supplementary Text

PAAM-TPMLH to PAAM-TPMLN hydrogel conversion

To verify the TPMLH to TPMLN conversion over different soaking steps, we performed FTIR measurements on initial and final samples, as well as samples from intermediate steps. Since the presence of water can greatly affect the FTIR measurements, the hydrogels were dried and ground for FTIR testing. All samples were taken from the same piece of hydrogel sheet. Additionally, as a reference, we measured the FTIR on pure TPMLH and TPMLN (Figure S1A). Since TPMLH and TPMLN share a similar molecular structure, most of their peaks will overlap. However, the characteristic peak between 2200-2300 wavenumbers can be used to distinguish the presence of TPMLN, while multiple small peaks between 2300-2400 wavenumbers can be used to distinguish the presence of TPMLH (Figure S1B). From the FTIR analysis, the peaks between 2300-2400 wavenumbers diminish in the final PAAM-TPMLN hydrogel sample, with a single peak formed between 2200-2300 wavenumbers. The FTIR results shown in Figure S1A and S1B indicate a successful conversion of TPMLH to TPMLN in the post-polymerization conversion reaction.

To verify the complete removal of the KCN after TPMLH to TPMLN conversion, we measure the KCN residual concentration in the disposed solution from the final hydrogel washing steps. We repeat the washing steps 4 times with deionized water. The hydrogel-to-washing solution weight ratio is about 1:100. In each washing step, the hydrogel thin sheet (1mm) is soaked in the washing solution under constant stirring for 1 hr, and taken out. The washing solution is then conditioned to pH 7, and the cyanide ion concentration is measured using cyanide ion testing strips (MilliporeSigma Quantofix/CTL VISOCOLOR ECO Cyanide). The estimated cyanide ion concentrations in the washing solution are: first wash >30 mg/L, second wash 0.2-1 mg/L, third wash 0.01-0.02 mg/L, fourth wash (< 0.01mg/L; trace).

Mechanical testing and modulus extraction

Following the synthesizing steps described in the method section, we prepared several PAAm-TPMLH, PAAm-TPMLN (Post converted) samples into 40x5x1 mm strips for mechanical testing. For comparison, we also prepared PAAm-TPMLN hydrogel using direct polymerization by directly dissolved TPMLN molecule into the prepolymer solution in place of TPMLH. Due to its limited solubility, it is only practical to incorporate 8 mM TPMLN in the same DMSO/DI water solvent (DMSO 92%vol) using this direct polymerization method. All samples are fully swollen in water in dark before tensile testing. Since the TPMLH is pH sensitive, to prevent excessive swelling, its swelling medium is pre-conditioned to pH 9.5 using NaOH. Those samples are subjected to uniaxial tensile testing and the shear modulus is extracted using incompressible Neo-Hookean model. The as prepared PAAm-TPMLH hydrogel has a shear modulus of 7.15±0.84 kPa. The PAAm-TPMLN hydrogel has a shear modulus of 7.45±1.53 kPa. The directly polymerized PAAm-TPMLN hydrogel has a shear modulus of 4.60±0.77 kPa. In comparison, before and after conversion, the PAAm-TPMLH/TPinN hydrogel shares a similar shear modulus and both show good stretchability (>100%) at 90 mM TPMLH/TPMLN concentration. (Figure S1) On the contrary, directly polymerized PAAm-TPMLN is significantly softer and less stretchable (<50%).

Spectrometer setup and absorption spectrum measurement

The spectrometer setup is assembled with a spectrometer (Spectral Product SM245), a low power hybrid light source (Spectral Product ASBP-DW-F-BAL), and a Cuvette housing (Thorlab CVH100). (Figure S3 A) Samples are prepared into flat disks and clamped inside a customized cuvette holder. (Figure S3 B,C)

As a proof to verify the TPMLN distribution inside the hydrogel sheet, we measured the absorption spectrum of 4 different cut out samples from the same piece of hydrogel (TPMLN = 0.090). Figure S2 d shows 4 absorption spectrums overlapping with each other, indicating the TPMLN concentration variation is minimal within a single piece of hydrogel.

Hydrogel light attenuation and through thickness gradient activation

In this section, we use the absorption spectrum to study light attenuation property of the hydrogel with a thin hydrogel section slice (Sample S5). Hydrogel sheets are first exposed to a low power UV source of 275nm and 310 nm. (0.2mW/cm2) It is then sectionally sliced and imaged using a Zeiss AxioScope A1 upright microscope with Zeiss AxioCam105 under tungsten-halogen bright field illumination. The color gradient is then extracted with ImageJ. (Figure S4)

AC impedance measurement and parameter extraction

In this section, we will show a complete set of impedance measurements along with fitting parameters extracted with ZView. Figure S5 shows the measurement setup. The sample holder has a pair of copper electrodes with adjustable separation distance. A hydrogel sample is clamped in between these two electrodes and connected to the LCR meter for impedance measurement. The UV LED is held above the sample and driven by a constant current power supply. (Figure S5)

The collected hydrogel frequency response is then fitting with model described in the main text in ZView to obtain the parameter for each component. Table S1 and Table S2 shows examples of fitting results for hydrogel samples with different TPMLN concentration before and after 275 nm, 295 nm, and 310 nm UV irradiation. Corresponding results are used for Figure 3 in the main text.

Design of the logic processing unit

The circuit diagram is described in Figure S6. Hydrogels are connected to the circuit through a pair of copper electrodes.

Design of the artificial retina

In this section, we will discuss the design of the artificial retina. The complete retina is made from molding and assembling. A total of 25 copper electrodes are made by photoresist-etching technique will be used for sensing electrode. (Figure S7 A) The sensing electrode and middle reference electrode are assembled together onto a 3D printed plastic base to form one single sensing unit. (Figure S7 B) A total of 25 sensing units are then assembled onto a 3D printed mold. The mold is then sealed and filled with silicone elastomer to encapsulate all sensing units. (Figure S7 C,D) After pulling the artificial retina sensory array out from the mold, the hydrogel can be installed in place for image sensing. (Figure S7 E)

The retina performance is first quantified using an isolated real-scale single pixel sensory unit. (Figure S8 A) The testing unit is fabricated using the same technique as the final retina. The only exception is the 3D printed resin bottom support being replaced with a square-shaped PVC block. The measured unit cell capacitance is also plotted against time while the UV LEDs are being turned on and off alternatively every 10s for 7 complete cycles (140 s). (Figure S8 B) The following Figure S8 shows the testing setup and signal readouts.

Fig. S1.

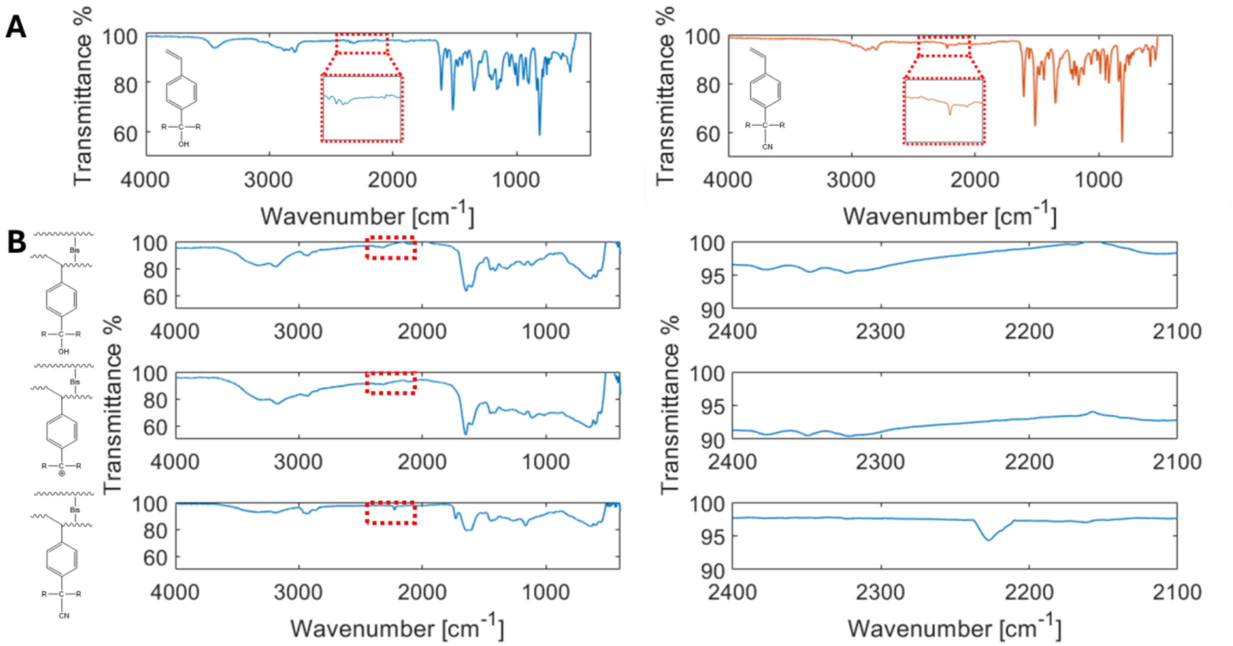

Fig. S1, FTIR for pure TPMLH, TPMLN, and PAAm-TPMLX samples. (**A**) FTIR measurements of TPMLH and TPMLN showing characteristic peaks (**B**) FTIR measurements of PAAM-TPMLH, PAAM-TPML+, PAAM-TPMLN samples.

Fig. S2.

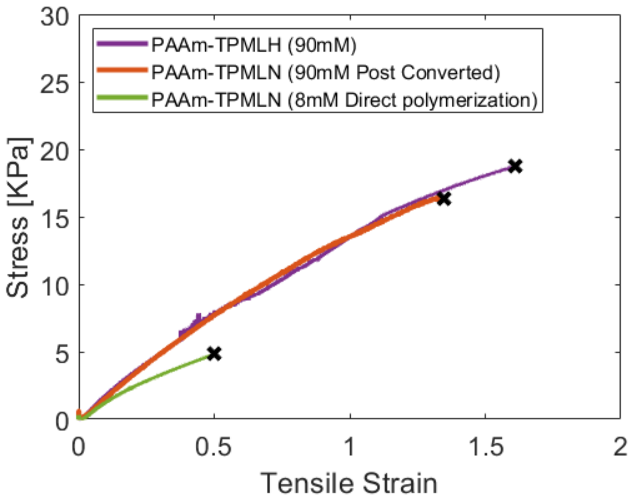

Fig. S2, Tensile testing results for PAAm-TPMLH samples and two differently prepared PAAm-TPMLN samples. Using the synthesizing procedure proposed in this paper, the hydrogel's mechanical property has been significantly improved.

Fig. S3.

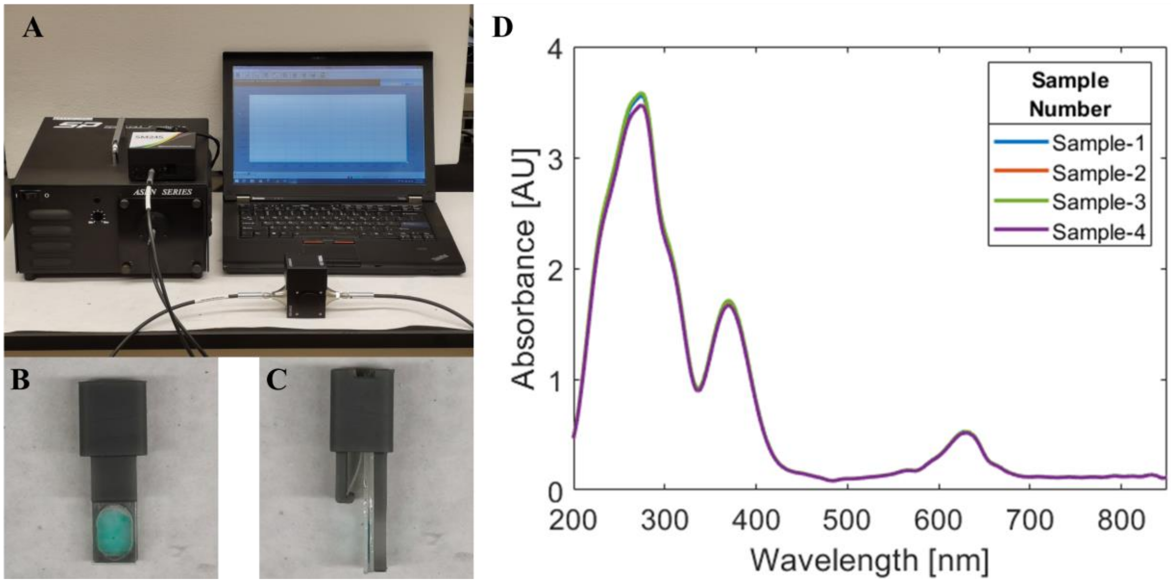

Fig. S3, Spectrometer setup and the customized cuvette holder. (**A**), Spectrometer setup. (**B, C**) A hydrogel sample of 200um thickness is inserted in between two quartz glass. The sample is then fitted in a customized cuvette holder to spectroscopy measurement. (**D**) Absorption spectrum of the 4 different samples cut-out from one hydrogel sheet at different locations.

Fig. S4.

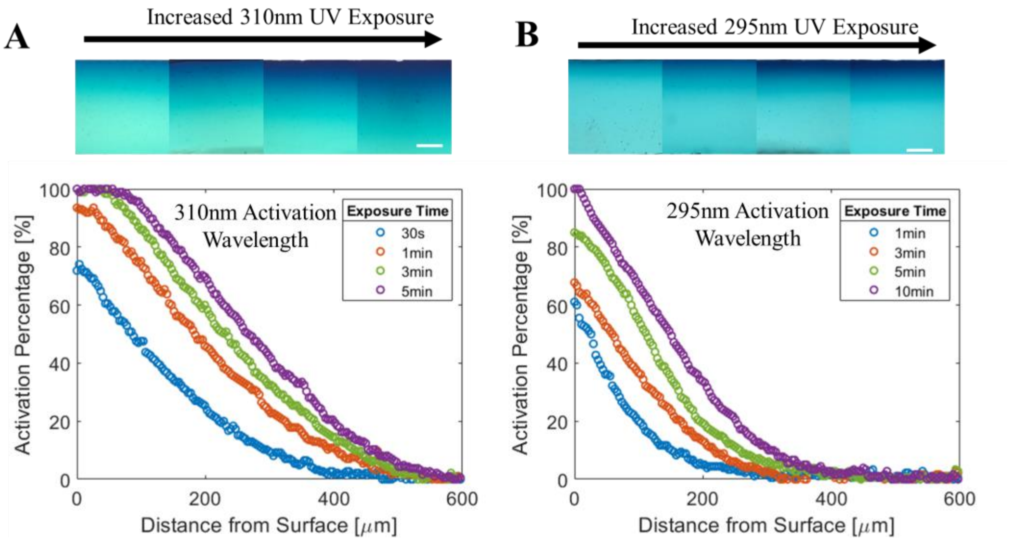

Fig. S4, Through thickness activation. (**A**) 310nm can achieve a more uniformed through thickness activation profile than (**B**) 295nm. Scale bar 200um.

Fig. S5.

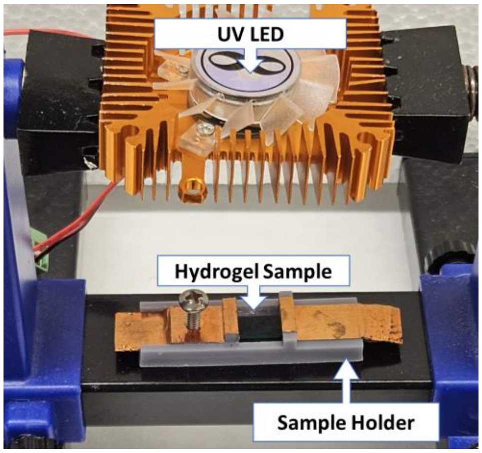

Fig. S5, Impedance measurement setup. The UV LED is fixed 5 cm above the sample. The hydrogel sample is clamped between two vertical copper electrodes where the copper electrodes are connected to an electrochemical workstation.

Fig. S6.

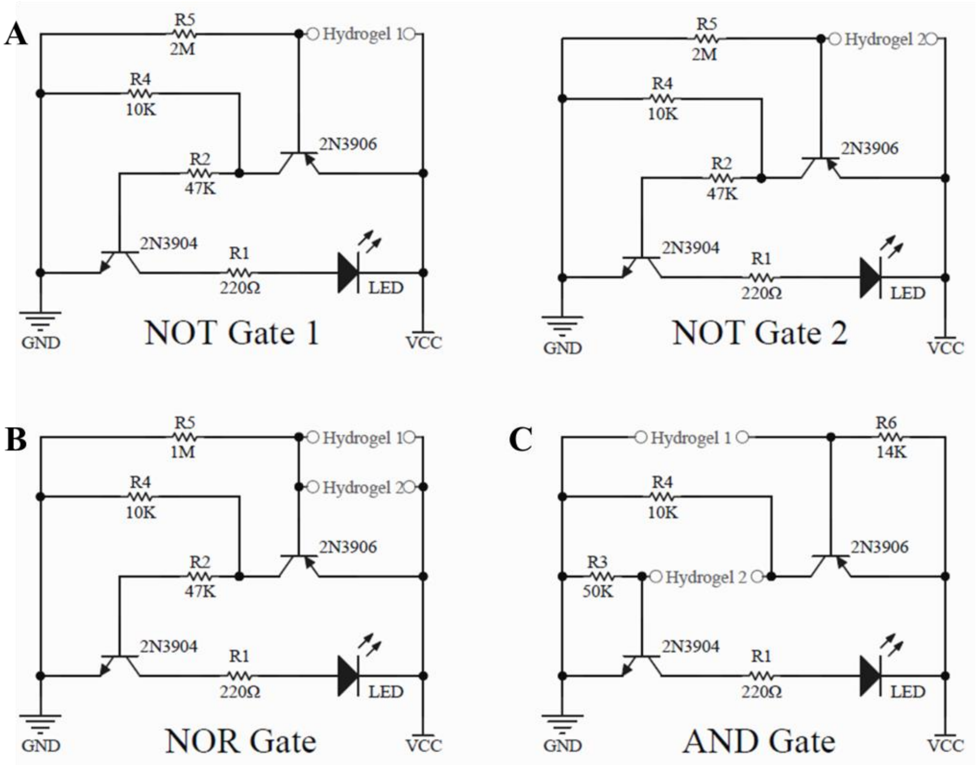

Fig. S6, The circuit diagram of three logic gates. (**A**) Circuit diagram for NOT gate with two hydrogel connections. (**B**) Circuit diagram for NOR gate with two hydrogel connections. (**C**) Circuit diagram for AND gate with two hydrogel connections. Hydrogels are connected to each circuit at the marked locations.

Fig. S7.

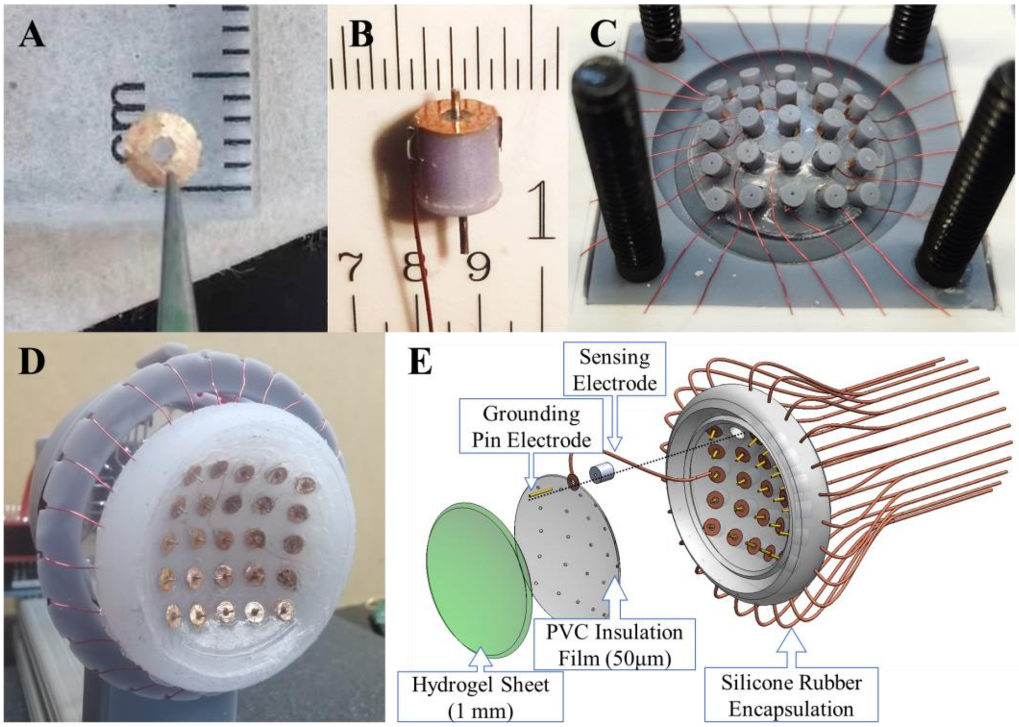

Fig. S7, The complete retina is made from molding and assembling. (**A**) Photo-etched copper washer sensing electrode. (**B**) Assembled single sensing unit. (**C**) Assembled 25 sensing units in a 3D printed mold. (**D**) The artificial retina sensory array after being pull out from the mold, before placing the hydrogel. (**E**) Exploded view of the complete artificial retina assembly.

Fig. S8.

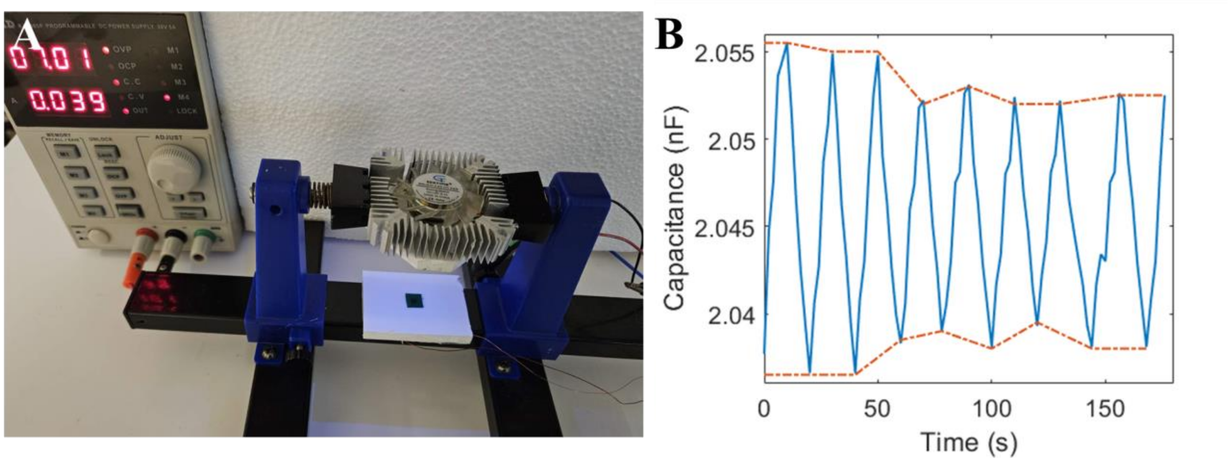

Fig. S8, Testing a single sensory unit for performance calibration. (**A**) The testing apparatus to calibrate single sensory unit. A single sensory unit with the same dimension and material from the retina sensory array are placed 5 cm away from the designated UV LED light source and (**B**) the capacitance change in response to light irradiation is measure using an electrochemical workstation.

Table S1.

Table S1 Example fitting parameters from ZView for hydrogel samples before UV activation.

UV Wavelength (nm)	[TPMLH] (mM)	R-Contact (Ohm)	R-Bulk (Ohm)	C-Bulk (F)	CPE-T	CPE-P
275	0	1.67E+02	1.01E+06	3.37E-13	5.90E-07	0.48
	5	2.24E+02	9.28E+05	3.69E-13	6.69E-07	0.47
	10	4.11E+02	5.35E+05	3.19E-13	8.81E-07	0.52
	30	3.38E+02	3.91E+05	4.47E-13	1.31E-06	0.50
	60	2.26E+02	2.90E+05	3.07E-13	3.66E-06	0.36
	90	2.79E+02	2.38E+05	3.40E-13	1.13E-05	0.21
295	0	2.85E+02	8.55E+05	9.42E-13	1.17E-05	0.37
	5	3.00E+02	6.83E+05	1.19E-13	1.46E-05	0.38
	10	2.38E+02	6.28E+05	1.29E-12	1.59E-05	0.37
	30	1.00E+02	6.04E+05	3.22E-13	5.91E-07	0.50
	60	4.99E+02	5.24E+05	3.64E-13	6.30E-07	0.52
	90	4.00E+02	4.17E+05	4.67E-13	8.56E-07	0.50
310	0	1.14E+02	8.55E+05	1.90E-13	4.17E-07	0.48
	5	2.65E+02	3.38E+05	2.11E-13	1.18E-07	0.55
	10	2.46E+02	4.28E+05	3.81E-13	3.44E-07	0.60
	30	3.12E+02	2.16E+05	3.70E-13	3.44E-08	0.79
	60	2.41E+02	3.23E+05	1.79E-13	2.07E-05	0.40
	90	4.53E+02	3.03E+05	1.93E-13	2.16E-05	0.31

Table S2.

Table S2 Example fitting parameters from ZView for hydrogel samples after UV activation.

UV Wavelength (nm)	[TPMLH] (mM)	R-Contact (Ohm)	R-Bulk (Ohm)	C-Bulk (F)	CPE-T	CPE-P
275	0	1.57E+02	1.01E+06	3.35E-13	5.75E-07	0.48
	5	1.90E+02	7.23E+05	4.73E-13	8.57E-07	0.47
	10	5.54E+02	2.35E+05	7.26E-13	2.00E-06	0.52
	30	3.45E+02	2.31E+05	7.58E-13	2.22E-06	0.50
	60	2.47E+02	1.10E+05	8.07E-13	9.64E-06	0.35
	90	2.86E+02	1.02E+05	8.77E-13	1.17E-05	0.33
295	0	2.79E+02	8.47E+05	9.51E-13	1.18E-05	0.37
	5	3.66E+02	2.92E+05	2.76E-13	3.42E-05	0.36
	10	1.65E+02	2.15E+05	3.81E-13	4.65E-05	0.37
	30	8.10E+01	1.25E+05	1.58E-12	3.01E-06	0.49
	60	6.25E+02	7.65E+04	2.47E-12	4.18E-06	0.52
	90	3.55E+02	5.30E+04	3.64E-12	6.50E-06	0.51
310	0	1.27E+02	8.46E+05	1.92E-13	4.20E-07	0.48
	5	2.37E+02	1.29E+05	5.53E-13	3.60E-07	0.54
	10	2.06E+02	1.23E+05	1.34E-12	1.68E-06	0.57
	30	3.65E+02	3.16E+04	2.46E-12	2.31E-07	0.80
	60	1.89E+02	3.03E+04	2.03E-12	3.64E-05	0.33
	90	4.20E+02	2.59E+04	2.44E-12	3.39E-05	0.39

Movie S1.

Video demonsration of two NOT gate using the optoionic hydrogel. These two NOT logic gate are realized using the same piece of hydrogel with two separately controlled light paths. The on-off state of each output LED is controlled by two light inputs.

Movie S2.

Video demonsration of a AND gate using the optoionic hydrogel. This AND logic gate is realized using the same piece of hydrogel with two separately controlled light paths. The on-off state of the output LED is controlled by two light inputs.

Movie S3.

Video demonsration of a NOR gate using the optoionic hydrogel. This NOR logic gate is realized using the same piece of hydrogel with two separately controlled light paths. The on-off state of the output LED is controlled by two light inputs.
